# MiR-25-3p promotes the proliferation of triple negative breast cancer by targeting BTG2

**DOI:** 10.1186/s12943-017-0754-0

**Published:** 2018-01-08

**Authors:** Hua Chen, Hong Pan, Yi Qian, Wenbin Zhou, Xiaoan Liu

**Affiliations:** Department of Breast Surgery, The First Affiliated Hospital of Nanjing Medical University, 300 Guangzhou Road, Nanjing, Jiangsu 210029 People’s Republic of China

**Keywords:** TNBC, miR-25-3p, Proliferation, BTG2, AKT, ERK

## Abstract

**Background:**

Triple-negative breast cancer (TNBC) is highly invasive and aggressive and lacks specific molecular targets to improve the prognosis. MiR-25-3p promotes proliferation of many tumors and its role and underlying mechanisms in TNBC remain to be well elucidated.

**Methods:**

Differential expression of miR-25-3p in TNBC was measured with quantitative real-time PCR (qRT-PCR) in both TNBC tissues and cell lines and was validated in the Cancer Genome Atlas (TCGA) database. The effects of miR-25-3p on proliferation, apoptosis capacity of TNBC were evaluated using Cell counting kit-8 (CCK-8), colony formation assay and Annexin V-FITC/PI analyses. The tumor growth in vivo was observed in xenograft model. Luciferase reporter assay, qPCR and western blot were performed to validate a potential target of miR-25-3p in TNBC. Involvement of the AKT and MAPK pathways was investigated by western blot.

**Results:**

MiR-25-3p was found to be upregulated in TNBC in tissues and cell lines. MiR-25-3p promoted TNBC cell proliferation in vitro and tumor growth in xenograft model, while suppression of miR-25-3p induced cell apoptosis. The luciferase reporter assay confirmed that B-cell translocation gene 2 (BTG2) might be a direct target of miR-25-3p, and its expression was negatively regulated by miR-25-3p. Moreover, inhibition of BTG2 expression accounted for the role of miR-25-3p in TNBC. Furthermore, BTG2 suppression might indirectly activate the AKT and ERK-MAPK signaling pathways to mediate the downstream effects of miR-25-3p.

**Conclusions:**

This study demonstrates that miR-25-3p promotes proliferation by targeting tumor suppressor BTG2 and may identify new diagnostic and therapeutic targets in TNBC.

**Electronic supplementary material:**

The online version of this article (10.1186/s12943-017-0754-0) contains supplementary material, which is available to authorized users.

## Background

Breast cancer is the most common malignancy in women nowadays with nearly 1.67 million incidences worldwide each year [1]. According to the expression status of hormone receptors and human epidermal growth factor receptor-2 (HER2), breast cancer consists of luminal A-like, luminal B-like, HER2-positive and triple-negative molecular subtypes respectively [2]. Triple-negative breast cancer (TNBC) is defined as the subtype lacking expression of estrogen and progesterone receptors (ER and PR) and HER2, and is highly invasive and aggressive [3]. Chemotherapy remains the standard treatment strategy for TNBC due to the absence of specific therapeutic targets and patients with TNBC may have increased risk of early tumor relapse [4]. It is urgent to discover new molecular targets to improve the relative poor prognosis of TNBC.

MicroRNAs (miRNAs) are a class of small non-coding RNAs, which are 21–24 nucleotides in length. MicroRNAs regulate gene expression via binding to the 3’untranslated region (3’UTR) of the target mRNAs resulting in post-transcriptional repression or degradation [5]. MiRNAs play important roles in a series of tumor biological processes, including tumor proliferation, differentiation, apoptosis, migration and invasion [6]. Dysregulated miRNAs may act as tumor suppressors or oncogenic miRNAs by targeting oncogenes or tumor suppressors [7].

MiR-25, along with miR-106b and miR-93, is a member of the cluster located in intron 13 of the MCM7 oncogene on chromosome 7q22.1 [8]. Previous studies report that miR-25 is associated with several tumor types, including gastric cancer, prostate cancer, liver cancer, colon cancer and anaplastic thyroid carcinoma [9–13]. It can be oncogenic or a tumor suppressor depending on different cancer types. In breast cancer, it is found that miR-106b~25 cluster could improve metastasis of human breast cancer cells and miR-93 could promote proliferation, migration, and invasion of breast cancer cells [14, 15]. However, as the isoform of mature miR-25, the biological role and underlying mechanisms of miR-25-3p in TNBC have not been well elucidated.

B-cell translocation gene 2 (BTG2) is the first identified gene of the BTG/TOB gene family [16]. As a tumor suppressor in many types of malignancies, BTG2 is involved in proliferation, cell cycle progression, apoptosis, and DNA damage repair [17–20]. BTG2 expression is downregulated in many human cancers, which is associated with poor prognosis in breast cancer patients [21]. Furthermore, BTG2 can also inhibit proliferation, invasion, and induce apoptosis in MDA-MB-231 breast cancer cells [22]. The relationship between BTG2 and miRNA in human cancer has attracted wide interests recently [23–25], and it has also been reported that miR-25 can directly bind BTG2 in NSCLC cells [26]. In TNBC, Powell et al. have found that BTG2 loss can enhance metastatic potential in patient-derived xenograft PDX models [27], while the relationship between BTG2 and miR-25-3p in TNBC remains unknown.

In this study, we investigated the biological role of miR-25-3p in TNBC progression and further identified BTG2 as a direct target of miR-25-3p, which may be a promising biomarker for TNBC patients.

## Methods

### Patient tissue specimens

Breast cancer tissues and adjacent normal tissues were collected from patients who underwent modified breast cancer radical mastectomy or breast conserving surgery in the First Affiliated Hospital of Nanjing Medical University without accepting any chemotherapy prior to tumor resection. All tissues were frozen in liquid nitrogen immediately and stored at −80 °C after surgical removal. The patients and their relatives provided written informed consent for their clinical information. This study was approved by the ethical committee of Nanjing Medical University.

### Cell lines and cell culture

All cell lines include MDA-MB-231, MCF-7, ZR-75-1 and MCF10A were obtained from the American Tissue Culture Collection (ATCC). Sum-1315 Cell line was provided by Stephen Ethier (University of Michigan). All cell lines were cultured in DMEM (Gbico, Detroit, MI, USA) medium supplemented with 10% fetal bovine serum (Gbico, Detroit, MI, USA) and antibiotics (100 units/ml penicillin G and 100 mg/ml streptomycin) in a 5% CO2 37 °C incubator.

### Quantitative real-time PCR (qRT-PCR)

Total RNA was extracted from tissues and cells using Trizol reagent (Invitrogen) following the manufacturer’s protocol. For miRNA, cDNA was specifically synthesized and miRNA was detected with the Hairpin-it™ miRNA qPCR Quantitation Kit (GenePharma, China). Relative expression level of hsa-miR-25-3p was normalized to U6. The primers were as follows: miR-25-3p, forward: 5’-CATTGCACTTGTCTCGGTCTGA-3′, reverse: 5’-GCTGTCAACGATACGCTACGTAACG-3’;U6,forward:5’-CTCGCTTCGGCAGCACA-3′, reverse: 5’-AACGCTTCACGAATTTGCGT-3′. For BTG2 mRNA expression analysis, first strand cDNA was synthesized by using Primescript RT reagent (Takara, Japan). BTG2 mRNA expression which was normalized to GAPDH, was detected with SYBR Green Master Mix Kit (Roche, USA) following manufacturer’s instruction. The primers were as follows: BTG2, forward: 5’-CATCATCAGCAGGGTGGC-3′, reverse: 5’-CCCAATGCGGTAGGACAC-3′; GAPDH, forward: 5’-TGCACCACCAACTGCTTAGC-3′, reverse:5’-GGCATGGACTGTGGTCATGAG-3′.The real-time PCR reactions were performed in the ABI StepOne Plus (Applied Biosystems, Foster City, CA, USA) and the relative expression was calculated using the 2^-ΔΔCT^ method. All procedures were performed in triplicate.

### Western blotting

Protein was extracted from breast cancer cells, separated on 10% SDS PAGE gel and then transferred to polyvinylidene difluoride (PVDF) membranes. The transferred membranes were blocked in 5% non-fat dry milk in TBST for 2 h and incubated with primary antibodies overnight at 4 °C. The membranes were then incubated for 2 h in secondary antibodies at room temperature. The primary antibodies used in this study were as follows: anti-BTG2, anti-AKT, anti-p-AKT, anti-ERK, anti-p-ERK (diluted1: 1000, Abcam), anti-Caspase3, anti-Cleaved Caspaes3, anti-GAPDH, anti-Actin (diluted 1:1000, Cell Signaling Technology). GAPDH and Actin were used as an internal control. Proteins were visualized using a detection system of enhanced chemiluminescence (ECL).

### Lentivirus production and transfection

The miR-25-3p mimics and inhibitor lentivirus were constructed by Genechem (Shanghai, China) to overexpress or knockdown miR-25-3p in breast cancer cells. The GV272 empty construct (miR-NC) served as a negative control. Target cells (2 × 10^5^) were infected with 1 × 10^6^ lentivirus transducing units in the presence of 10μg/ml polybrene (Genechem, Shanghai, China). The lentiviral vector containing BTG2 DNA sequence and siRNA for BTG2 were constructed by Genepharma (Shanghai, China). When MDA-MB-231 and Sum-1315 cells grew to 40–50% confluence, cells were infected with lentiviral vectors miR-NC, miR-25-3p mimics and inhibitor respectively. The lentiviral vectors were transfected into BC cells with the multiplicity of infection (MOI) of 20 to the MDA-MB-231 and Sum-1315. Stable cell lines were selected by using 3μg/ ml puromycin (Sigma, USA) for 1 week.

### Luciferase reporter assay

The wild-type (WT 3’-UTR) or mutant (Mut 3’-UTR) miR-25 binding site in the 3’-UTR of BTG2 were synthesized and sub-cloned into the GV272 reporter vector (GV272: SV40-Luciferase-MCS-Poly A was purchased from Shanghai Genechem Co, Ltd.). Cells were transfected with appropriate plasmid and miR-25 duplex. Luciferase assays were done using the Dual-luciferase reporter assay system (Promega, USA) 48 h after transfection. Normalized luciferase activity was reported as Luciferase activity/Renilla Luciferase activity.

### Cell proliferation assay

Cell proliferation assays were conducted by using Cell Counting Kit-8 (CCK-8), (Beyotime, Shanghai, China) according to the manufacturer’s instructions. 2000 cells were seeded into each well of 96-well plate with 100ul DMEM supplemented with 10% FBS. At the indicated time point of everyday, the medium was exchanged by 110ul DMEM with CCK-8 (100ul DMEM and 10ul CCK-8) and the cells were incubated for 2 h. Then we measured the absorbance for each well at a wavelength of 450 nm (OD value) using an auto-microplate reader. Average OD values were used to estimate the number of cells of each group.

### Colony formation assay

Cell colony formation ability was measured by plate colony formation assay. 500 cells were added to each well of a 6-well plate and incubated for about 2 weeks until colony was obviously formed. Then the plate was gently washed and stained with crystal violet. The number of colonies was counted by observing the proliferation of single cell.

### EdU incorporation assay

The assay was performed using the EdU assay kit (RiboBio, China) following the manufacturer’s protocol. Cells were plated into 6-well plates (5 × 10^4^ cells/well) and cultured with DMEM (10% FBS) for 24 h. Cells were then incubated in EdU (50 μM) for 2 h at 37 °C and fixed in 4% formaldehyde for 30 min. After permeabilization with 0.5% TritonX-100 for 10 min, the cells were reacted with Apollo reaction cocktail (400uL) for 30 min. Subsequently, the nuclei were stained with DAPI for 30 min and visualized under a laser scanning confocal microscopy (Nikon, Japan).

### Cell apoptosis analysis

The cell apoptosis rate of MDA-MB-231 and Sum-1315 cells was analyzed after transfection with the miR-25-3p inhibitor and negative control. Cells were collected after washing twice with PBS, staining with the Annexin V-FITC/PI Apoptosis Detection Kit (BD Biosciences) and they were then evaluated by flow cytometry (BD Biosciences, Bedford, MD, USA).

### Tumor xenograft model in vivo

All animal experiments were approved by the NJMU Institutional Animal Care and Use Committee. A total of 20 female nude mice (BALB/c nude mice, Vitalriver, Nanjing, China; 6 weeks old) were randomly divided into 4 groups. Sum-1315-NC, Sum-1315-miR-inhibitor; Sum-1315-NC, Sum-1315-miR-mimics. Stably transfected cells were inoculated subcutaneously into the flank of nude mice. Tumors were measured with vernier calipers every 4 days, and the mice were euthanized after 3 weeks. The volume of the implanted tumor was calculated by using the formula: volume = (width^2^ × length)/2.

### Statistical analysis

Every experiment was repeated at least three times. All data was analyzed using SPSS 22.0 and presented as mean ± standard deviation (SD) or as indicated. The data was analyzed using two-tailed Student’s t-test for average differences. The expression data for paired samples was analyzed with Wilocoxon rank text. *P* values less or equal than 0.05 was considered to be statistically significant.

## Results

### MiR-25-3p is over-expressed in both TNBC tissue samples and cell lines

**Fig. 1:**
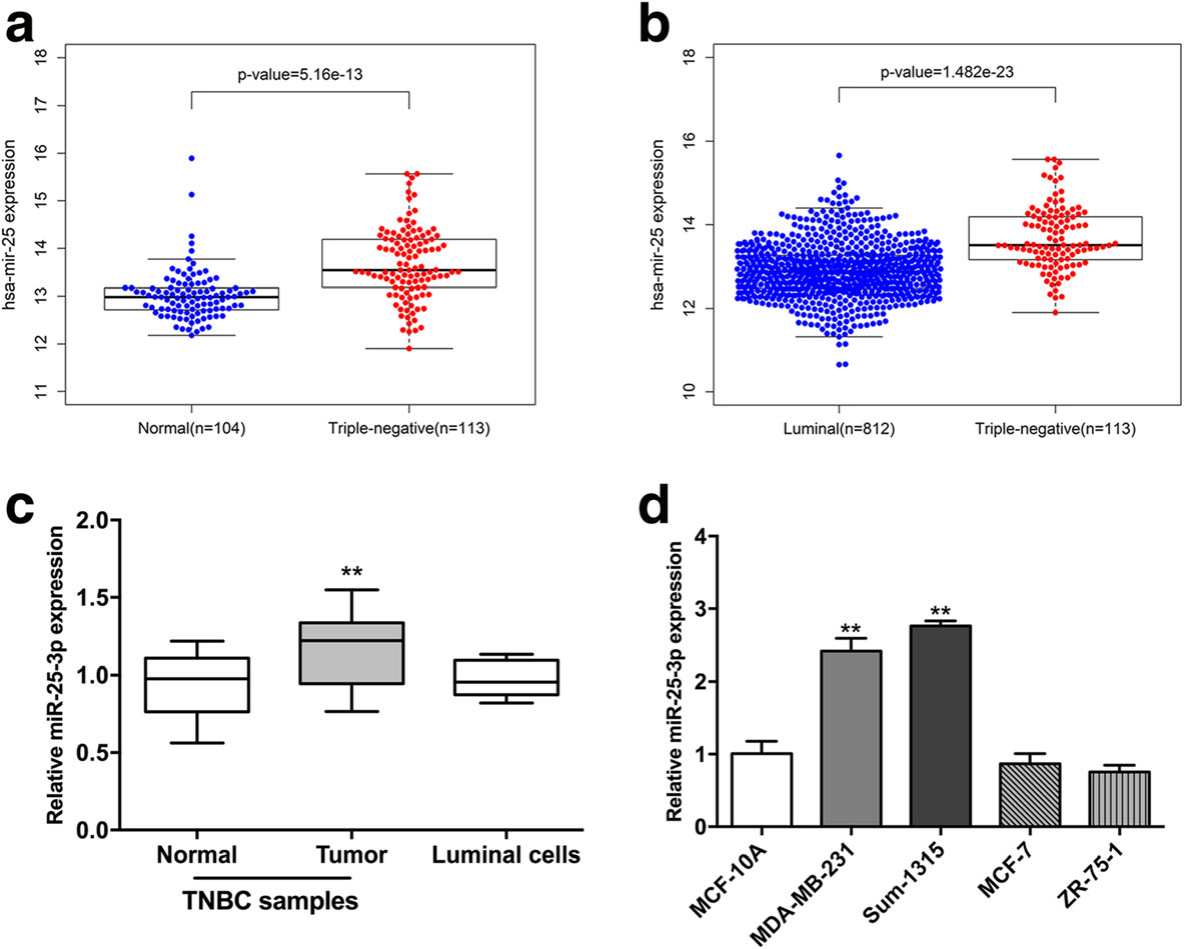
miR-25-3p was upregulated in TNBC tissues and cell lines. **a**, **b**. MiR-25 expression in TNBC samples as compared to normal breast samples and luminal breast cancer samples from TCGA database. **c**. The expression levels of miR-25-3p in 20 pairs of human TNBC tissues and adjacent normal tissues by qRT-PCR. **d**. The expression levels of miR-25-3p in TNBC cell lines and non-TNBC breast cancer cells. *p < 0.05, **p < 0.01. The data expressed as the mean ± SD

To identify differentially expressed miRNAs in TNBC, we performed miRNA microarray assay to examine the miRNA expression profiles of five paired samples of TNBC and adjacent normal tissues. Among the 9 differentially expressed miRNAs, miR-25-3p was upregulated by more than 2-folds in TNBC (Additional file 1). MiR-25 expression from the TCGA database was available for 113 triple negative tumors, 812 luminal tumors and 104 normal breast samples respectively. MiR-25 expression was higher in TNBC samples as compared to normal breast samples and luminal breast cancer samples (Fig. 1a and b). Besides, no significant difference was made between luminal tumor and normal samples. The expression level of miR-25-3p was further examined in 20 pairs of TNBC tissues and adjacent normal tissues, including luminal cell lines by quantitative real-time PCR, and was found to be significantly increased in TNBC tissues (Fig. 1c). In addition, miR-25-3p level was also found to be over-expressed in TNBC cell lines (MDA-MB-231, Sum-1315) compared with luminal breast cancer cells (MCF-7, ZR-75-1) and non-tumorigenic MCF10A cells, consistent with the results from clinical TNBC samples. (Fig. 1d).

### MiR-25-3p promotes TNBC cell proliferation in vitro

**Fig. 2:**
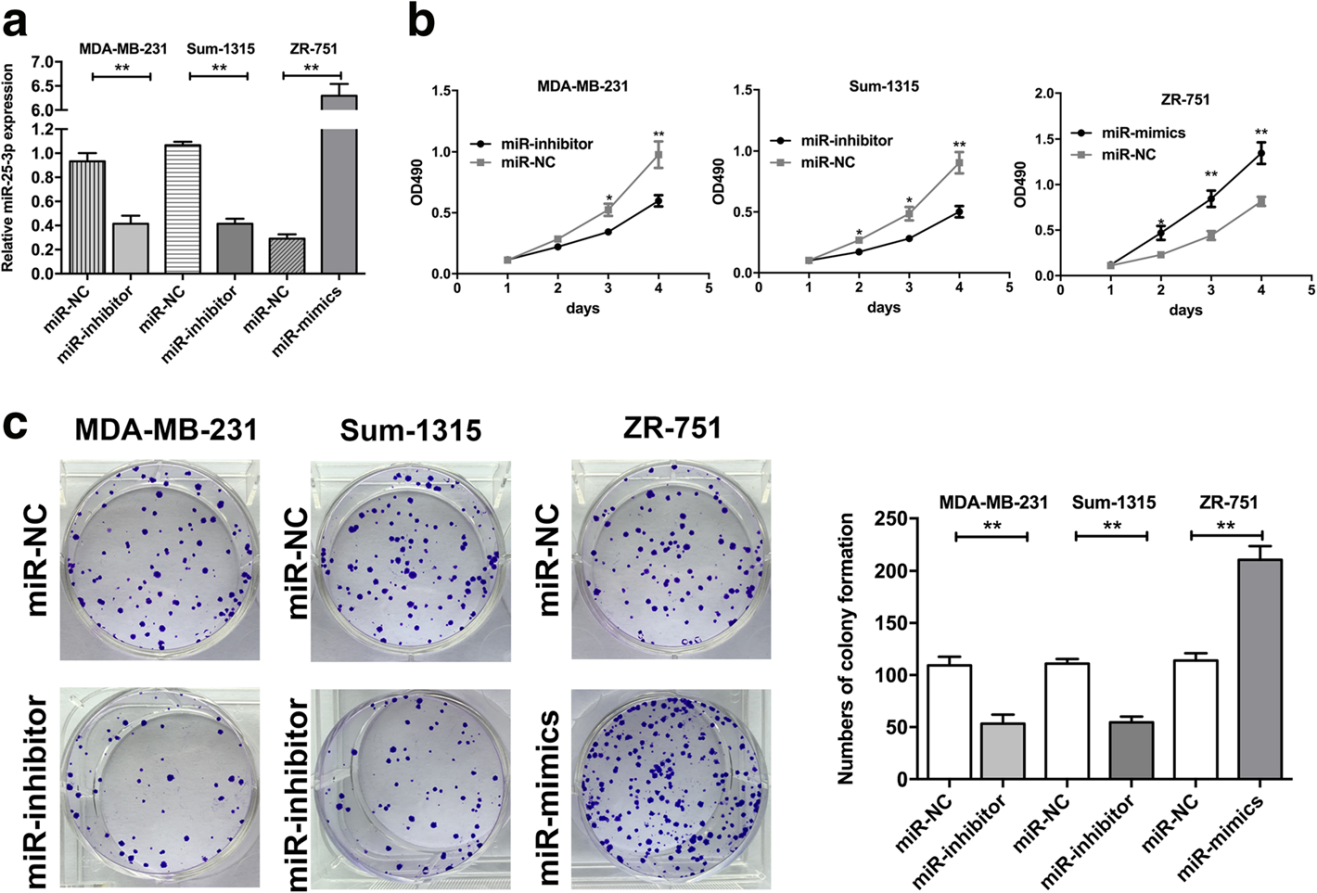
miR-25-3p promoted proliferation of TNBC cells in vitro. **a**. qRT-PCR was used to verify the expression of miR-25-3p in cells transfected with mimics and inhibitor lentivirus respectively. **b**. Cell proliferation was determined by CCK-8 assays in MDA-MB-231, Sum-1315 and ZR-751 cells transfected with miR-25-3p mimics and inhibitors lentivirus. **c**. The colony formation results of cells transfected with mimics and inhibitors lentivirus. *p < 0.05, **p < 0.01. The data expressed as the mean ± SD

To further investigate the role of miR-25-3p in triple-negative breast cancer. MDA-MB-231 and Sum-1315 cells were transfected with miR-25-3p mimics and inhibitor lentivirus respectively, meanwhile luminal cells ZR-751 were transfected with miR-25-3p mimics lentivirus. The transfection efficacy of miR-25-3p into TNBC and luminal cells was verified by qRT-PCR (Fig. 2a and Additional file 2). CCK-8 assay was used to examine the effect of miR-25-3p on the proliferative ability of BC cells. The results revealed that growth rate of MDA-MB-231, Sum-1315 and ZR-751 cells transfected with miR-25-3p mimics was significantly increased compared with negative control, while the cells transfected with miR-25-3p inhibitor showed the opposite effect (Fig. 2b and Additional file 2). Consistently, colony formation assay showed that over-expression of miR-25-3p could promote TNBC cell proliferation, whereas inhibition of miR-25-3p suppressed the effects (Fig. 2c and Additional file 2).

### Inhibition of miR-25-3p reduces DNA replication and induces apoptosis in TNBC

**Fig. 3:**
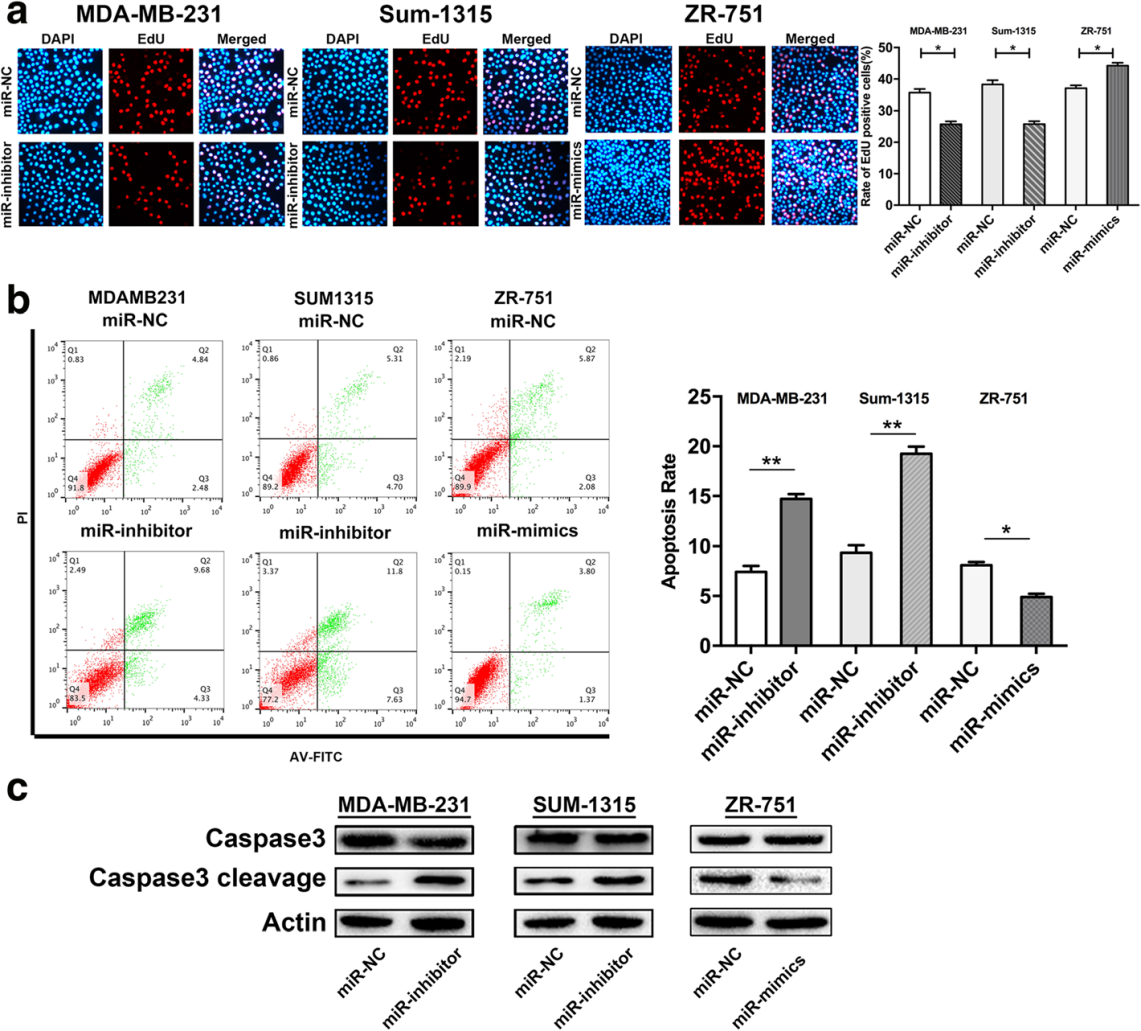
Inhibition of miR-25-3p reduced DNA replication and induced apoptosis in TNBC cells. **a**. Edu cell growth in MDA-MB-231, Sum-1315 and ZR-751 cells after transfection with miR-25-3p-inhibitor and miR-25-3p-mimics respectively compared with the control. **b**. Flow cytometry analysis of the effect of miR-25-3p expression alteration on cell apoptosis. **c**. Effects of miR-25-3p alteration on the apoptotic marker expression. *p < 0.05, **p < 0.01. The data expressed as the mean ± SD

The EdU incorporation assay was performed to examine the effect of miR-25-3p on DNA replication, as a more specific evaluation of proliferation. MDA-MB-231 and Sum-1315 cells transfected with the miR-25-3p inhibitor revealed a significantly decreased EdU-positive cells compared with control group, while TNBC cells and ZR-751 cells with the miR-25-3p mimics increased the positive rate. (Fig. 3a and Additional file 3).

We further assessed the role of miR-25-3p in regulating apoptosis of TNBC cells by flow cytometer. As shown in Fig. 3b, miR-25-3p inhibition induced apoptosis in MDA-MB-231 and Sum-1315 cell at 48 h compared with the negative control, and overexpression of miR-25-3p in ZR-751 and TNBC cells could reduce the effects (Additional file 3). Next, western blot analysis was performed to examine caspase 3 and caspase 3 cleavage in miR-25-3p inhibited cells. Consistently, the data showed that miR-25-3p inhibition obviously upregulated the apoptotic marker expression of caspase 3 cleavage and miR-25-3p overexpression decreased it (Fig. 3c and Additional file 3). Our results indicated that miR-25-3p suppression reduced DNA replication and induced cell apoptosis in TNBC cells.

### MiR-25-3p promotes tumor growth of TNBC cells in vivo

**Fig. 4:**
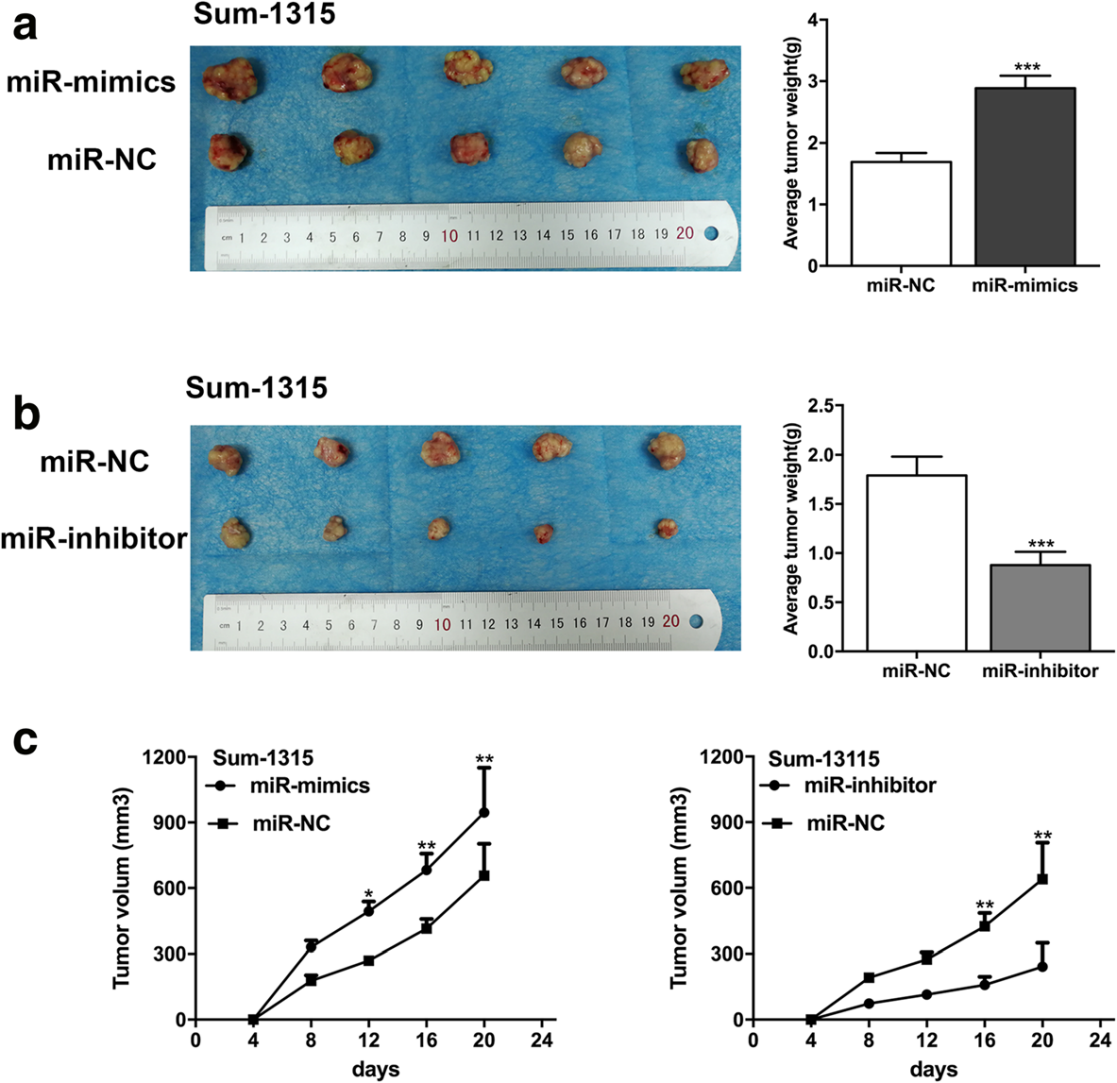
miR-25-3p promoted tumor growth of TNBC cells in vivo. **a**, **b**. Tumors were obtained from nude mice injected subcutaneously with Sum-1315 cells transfected with miR-25-3p mimics and inhibitor and tumor weights were measured respectively. **c**. The growth curves were determined by measuring tumor volumes every 4 days. *p < 0.05, **p < 0.01, ***p < 0.001. The data expressed as the mean ± SD

In order to investigate the effects of miR-25-3p expression on tumor growth in vivo, Sum-1315 cells transfected with miR-25-3p mimics or inhibitor lentivirus, or with negative control were injected subcutaneously into nude mice xenograft model respectively. Compared with the negative control, tumor weight showed a significantly increase in miR-25-3p mimics group. Consistent with in vitro cell growth results, tumor growth rate was remarkably higher in the miR-25-3p mimics group than in the negative control group. However, tumors in miR-25-3p inhibitor group showed the opposite effects (Fig. 4a-c).

### BTG2 is a direct target of miR-25-3p

**Fig. 5:**
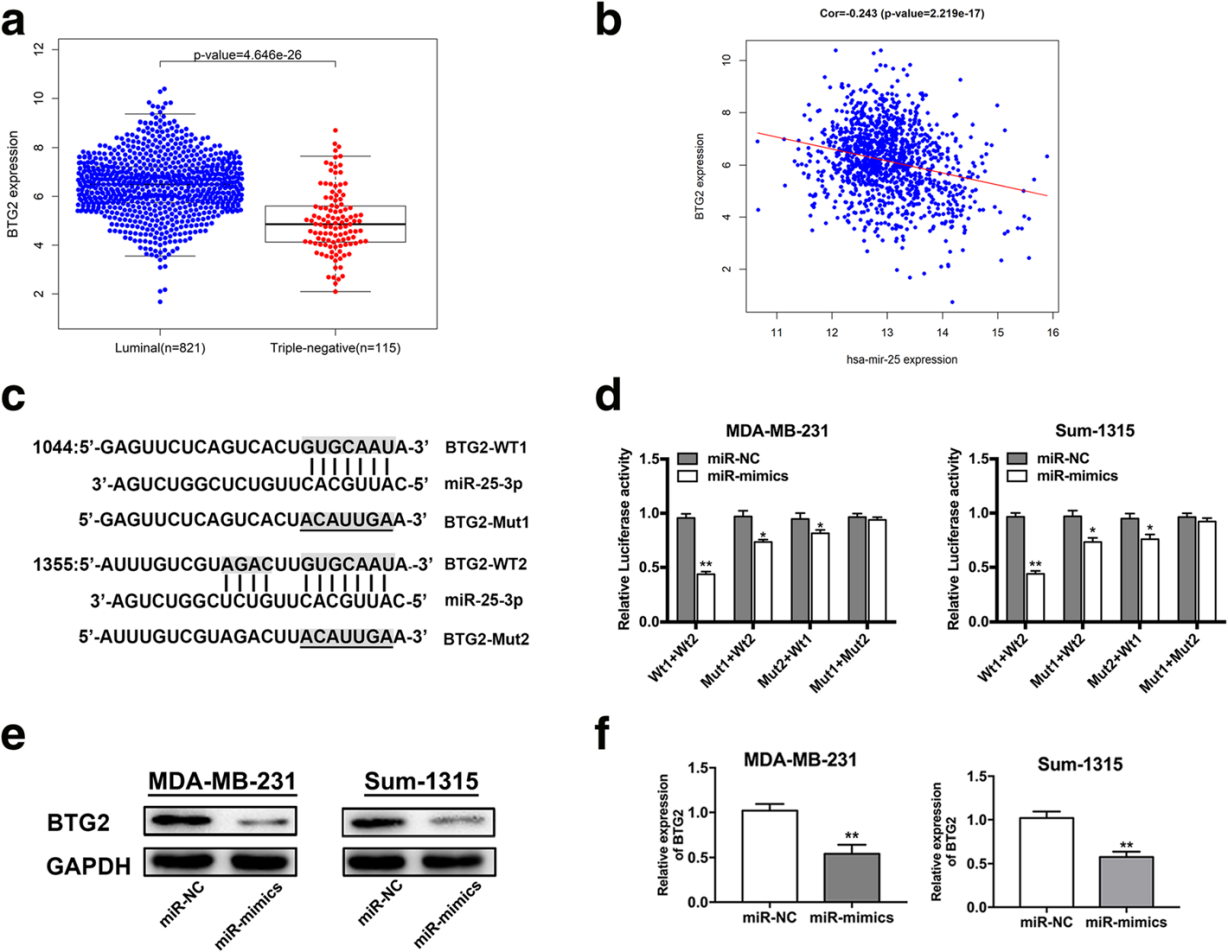
BTG2 is a direct target of miR-25-3p. **a**. BTG2 expression is suppressed in triple negative breast cancer samples when compared to luminal breast samples. **b**. Negative correlation between the expression levels of miR-25 and BTG2 in breast cancer specimens from TCGA database (*r* = −0.243, *P* < 0.05). **c**. The seed sequence of miR-25-3p is complementary to the 3’UTR of BTG2. **d**. Luciferase activity was analyzed in cells co-transfected with miR-25-3p mimics or negative control with GV272-BTG2 or GV272-BTG2-mut1/2. **e**. Western blot analysis showing suppression of BTG2 protein levels in TNBC cells transfected with miR-25-3p mimics. **f**. Expression of BTG2 mRNA in TNBC cells after miR-25-3p expression alteration by qRT-PCR. *p < 0.05, **p < 0.01. The data expressed as the mean ± SD

To explore the underlying mechanism of miR-25-3p, we searched the miRNA bioinformatics prediction websites (Targetscan, PicTar and miRanda), and identified BTG2 as a potential target of miR-25-3p. BTG2 is considered as a tumor suppressor gene in a variety of tumors, and it has been reported that BTG2 inhibits proliferation, invasion, and induces apoptosis of triple-negative breast cancer cells [22]. In TCGA database, BTG2 expression was available for 115 TNBC and 821 luminal breast cancer samples and it was lower in TNBC samples compared with lumial breast cancer samples (Fig. 5a). It was also found that there was a negative correlation between the expression levels of miR-25 and BTG2 in breast cancer specimens at the mRNA level from TCGA database (Pearson’s correlation, *r* = −0.243, *P* < 0.05) (Fig. 5b). We further performed a dual-luciferase reporter assay to identify whether BTG2 was a direct target of miR-25-3p in breast cancer. We cloned the wild-type BTG2 3’UTR fragment with binding sites (BTG2-wt1/2) and 3’UTR fragment with mutated sequence (BTG2-mut1/2) into separate GV272 luciferase reporter vectors (Fig. 5c). MiR-25-3p mimics or negative control plasmid was co-transfected with the BTG2-wt1/2 or BTG2-mut1/2 respectively. MiR-25-3p was shown to significantly decreased luciferase activity of the wild-type BTG2 3’UTR in MDA-MB-231 and Sum-1315 cells, while mutation at either of the binding sites could attenuate the effect and the activity did not show difference when transfected with both BTG2-mut1 and mut2 vector (Fig. 5d). The results indicated that co-transfection of miR-25-3p and BTG2 could remarkably repress the luciferase activity in TNBC cells. As shown in the western blot analysis, BTG2 protein expression decreased in miR-25-3p mimics groups of MDA-MB-231 and Sum-1315 cells (Fig. 5e). BTG2 mRNA level also decreased in MDA-MB-231 and Sum-1315 cells transfected with miR-25-3p mimics compared with the negative control groups envidenced by qRT-PCR (Fig. 5f). Overall, it suggested that BTG2 gene might be a direct target of miR-25-3p and that its expression was negatively regulated by miR-25-3p.

### Inhibition of BTG2 expression accounts for miR-25-3p function in TNBC

**Fig. 6:**
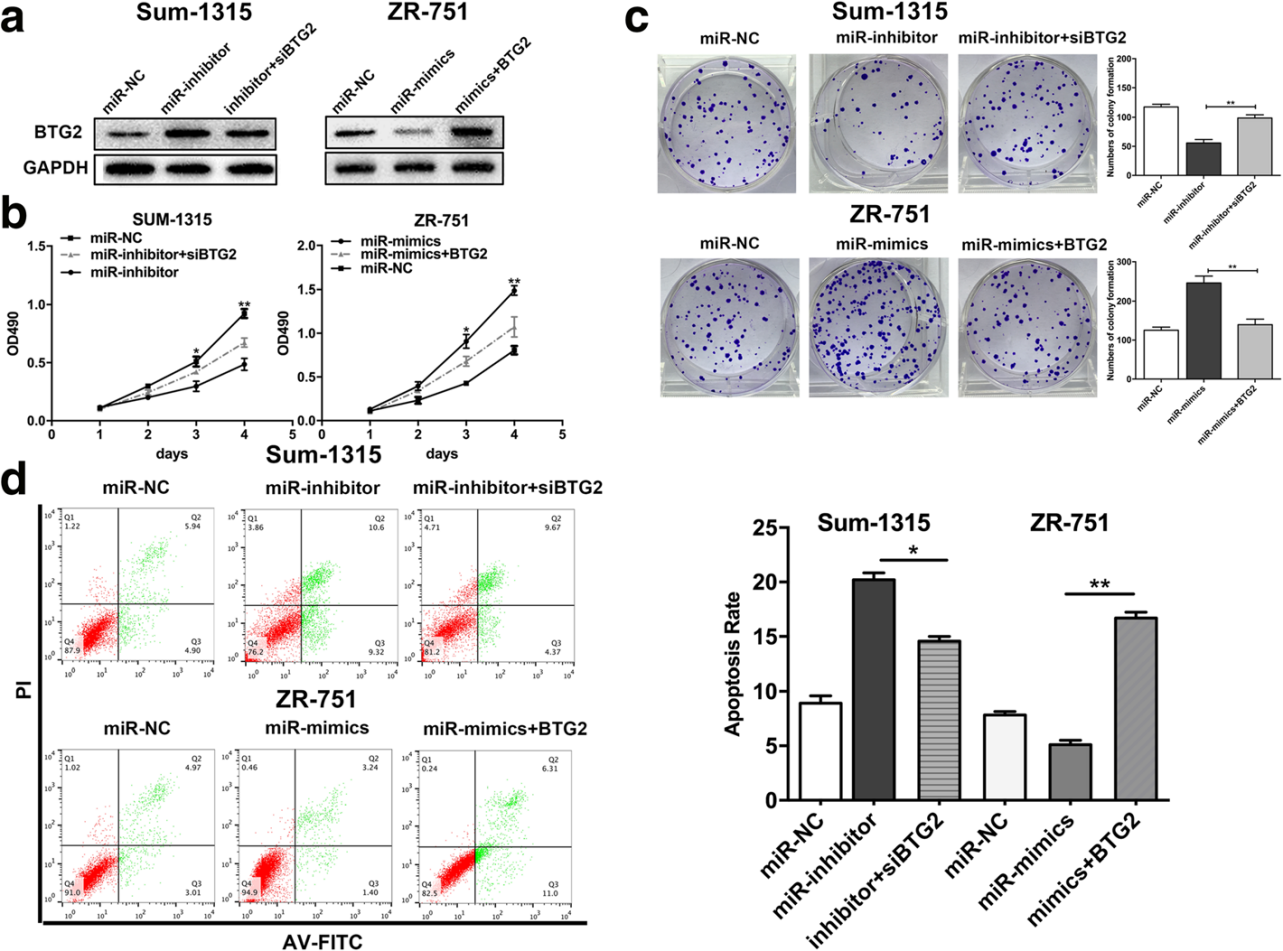
Inhibition of BTG2 expression accounted for function of miR-25-3p in TNBC. **a**. BTG2 protein expression level was measured by western blot. **b**, **c**. cell proliferation of Sum-1315 and ZR-751 cells transfected with GV272 empty construct (miR-NC), miR-25-3p inhibitor lentivirus (miR-inhibitor) and BTG2 interfering RNA (siBTG2),miR-25-3p mimics lentivirus (miR-mimics) and BTG2 lentiviral vector(lv-BTG2) was determined by CCK-8 and colony formation assay. **d**. The cell apoptosis of Sum-1315 and ZR-751 cells after cotransfection was measured in flow cytometry analysis. *p < 0.05, **p < 0.01. The data expressed as the mean ± SD

To further verify whether the effects of miR-25-3p on TNBC was mediated by BTG2, BTG2 was upregulated by transfection with miR-25-3p inhibitor lentivirus, and silenced by transfection with BTG2 interfering RNA (siRNA) in Sum-1315 cells; In ZR-751 cells, it was downregulated by transfection with miR-25-3p mimics lentivirus, and overexpressed with BTG2 lentiviral vector. As expected, the miR-25-3p inhibitor increased BTG2 protein levels, and this effect was reversed by BTG2 siRNA, while miR-25-3p mimics decreased BTG2 protein levels, which reversed by LV-BTG2 (Fig. 6a). Through CCK8 assay and colony formation assay, it was found that inhibition of miR-25-3p significantly decreased cell growth rate and BTG2 siRNA partially reversed the effects. In addition, over-expression of BTG2 could reverse the promoting effect of miR-25-3p (Fig. 6b-c). Similarly, BTG2 siRNA could significantly reduce the apoptosis rate in Sum-1315 cells transfected with miR-25-3p inhibitor. In ZR-751 cells transfected with miR-25-3p mimics, BTG2 could partially induce apoptosis (Fig. 6d). Taken together, it suggests that miR-25-3p acts as an oncogenic miRNA in TNBC partially by suppression of BTG2 and BTG2 may be the functional target of it.

### Downregulation of miR-25-3p attenuates the activation of AKT and ERK MAPK pathway through BTG2

**Fig. 7:**
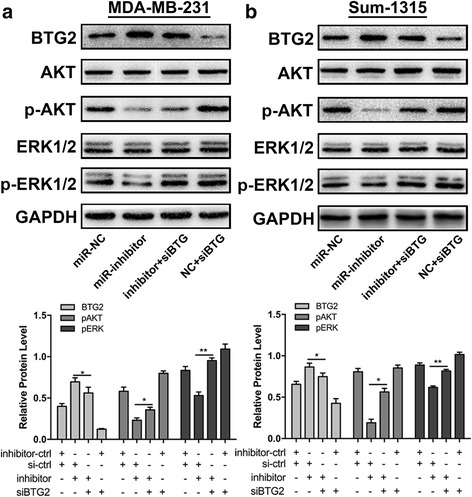
miR-25-3p functioned through BTG2 in AKT and ERK MAPK signaling pathway. **a**, **b**. western blot for BTG2, AKT, p-AKT, ERK1/2 and p-ERK1/2 in MDA-MB-231 and Sum-1315 cells transfected with inhibitor-control (inhibitor-ctrl) and siBTG2 scrambled oligonucleotide (si-ctrl), inhibitor and si-ctrl, inhibitor and siBTG2, or inhibitor-ctrl and siBTG2. Protein expression was quantified by band intensity and normalized to GAPDH. *p < 0.05, **p < 0.01. The data expressed as the mean ± SD

It has been reported that BTG2 is a negative regulator of AKT and ERK MAPK signaling pathways, which play the key role in cell proliferation and apoptosis [28–30]. In order to explore the mechanisms how miR-25-3p and BTG2 promote the proliferation and induce the apoptosis in TNBC, expression of BTG2 and downstream proteins of AKT and ERK signaling pathways were determined by western blot, followed by transfection of miR-25-3p inhibitor or BTG2 interfering RNA (siRNA) into MDA-MB-231 and Sum-1315 cells. As we expected, suppression of BTG2 activated the expression of p-AKT and p-ERK1/2 levels, whereas miR- 25-3p inhibition was found to attenuate the activation. Moreover, BTG2 siRNA restored p-AKT and p-ERK1/2 activities in miR-25-3p inhibited cells (Fig. 7a-b). These data further suggest that BTG2 is a downstream functional regulator of miR-25-3p through AKT and ERK signaling pathway.

## Discussion

TNBC is a very aggressive and heterogeneous tumor subtype with relatively poor prognosis and lacks effective targeted therapy. It is urgent to identify targets to provide possible diagnosis and therapeutic strategy. Aberrant expressions of miRNAs act as tumor suppressors or oncogenic factors in the development and progression of different cancers [7]. In this study, miRNA microarray analysis was performed on TNBC and adjacent normal tissues and we identified 9 differentially expressed miRNAs. We confirmed that miR-25-3p expression was upregulated in both TNBC tissue samples and cell lines compared to adjacent normal tissues and luminal cells and this finding was validated in TCGA database.

Previous studies have described the role of miR-106b~25 cluster in breast cancer, Zhou et al. found that miR-106b~25 cluster negatively regulated EP300 to function in drug resistance, cell migration and invasion [31]. Smith showed the oncogenic role of miR-106b-25 through the Six 1 regulation [14]. However, very little was known about miR-25-3p and its biological effects on TNBC. We explored it and found that inhibition of miR-25-3p significantly suppressed TNBC cell proliferation, increased cell apoptosis in vitro, and reduced tumor growth in a xenograft model. Taken together, these results indicate that miR-25-3p acts as an oncogenic miRNA exerting an important effect on TNBC progression.

BTG2 has been identified as a tumor suppressor of the BTG/TOB gene family [32], which inhibits cell proliferation, invasion and promotes apoptosis in several tumors, including TNBC [22]. BTG2 is also closely associated with other tumor suppressor genes, such as RB, p53 and p73 [27, 33, 34]. Reduced expression of BTG2 is found to be related to tumor size, grade, metastasis, recurrence and poor survival in patients with breast cancer [21, 35]. Recently, He et al. have reported that miR-25 directly targets BTG2 and suppresses its expression in NSCLC [26]. In this study, we found that BTG2 could be a direct target of miR-25-3p in TNBC. BTG2 expression negatively correlated with miR-25-3p expression, where BTG2 expression was downregulated while miR-25-3p was upregulated in TNBC samples compared to normal and luminal breast cancer samples. We confirmed that miR-25-3p down-regulated BTG2 expression post-transcriptionally through binding to the 3’UTR of BTG2 mRNA by the luciferase reporter assay. Moreover, BTG2 suppression partially attenuated the effects of miR-25-3p inhibitor on cell proliferation and apoptosis.

In addition, our data also suggested that miR-25-3p/BTG2 axis regulated TNBC progression by involving in the AKT and ERK-MAPK signaling pathways. It has been reported that AKT and ERK-MAPK signaling pathway play a key role in cell proliferation and apoptosis [28, 36–38], and BTG2 is found to be a negative regulator of them [25, 29, 30]. Further study showed that miR-25-3p inhibitor attenuated activation of the signaling pathways and the inhibitory effect could be reversed by BTG2 interfering RNA (siRNA). Therefore, we considered that miR-25-3p activated the signaling pathway by degradation of BTG2 in TNBC cells. However, the specific mechanisms underlying the activation remains poorly understood and further investigations are needed.

## Conclusion

In summary, we have identified for the first time that the oncogenic miR-25-3p directly targets BTG2 in TNBC. Further studies revealed that the biological effects of miR-25-3p on TNBC cell proliferation and apoptosis were mediated through regulation of BTG2 and subsequent activation of AKT and ERK-MAPK signaling pathway. These findings may represent a promising diagnostic biomarker and a potential target therapeutic strategy for TNBC patients.

## Additional file

Additional file 1: Table S1. Nine differentially expressed miRNAs from miRNA microarray assay in TNBC and adjacent normal tissues. (DOCX 126 kb)
Additional file 2: FigureS1. a. qRT-PCR was used to verify the expression of miR-25-3p in MDA-MB-231 and Sum-1315 cells transfected with mimics b. Cell proliferation was determined by CCK-8 assays in MDA-MB-231, Sum-1315 cells transfected with miR-25-3p mimics. c. The colony formation results of cells transfected with mimics lentivirus. **p* < 0.05, ***p* < 0.01. The data expressed as the mean ± SD. (TIFF 1901 kb)
Additional file 3: Figure S2. a. Edu cell growth in MDA-MB-231, Sum-1315 after transfection with miR-25-3p-mimics compared with the control. b. Flow cytometry analysis of the effect of miR-25-3p expression alteration on cell apoptosis and apoptotic marker expression. **p* < 0.05, ***p* < 0.01. The data expressed as the mean ± SD. (TIFF 3545 kb)

## Abbreviations

BTG2: B-cell translocation gene 2
CCK: Cell counting kit
DAPI: 4′,6-diamidino-2- phenylindole
ER: Estrogen receptors
ERK: Extracellular regulated protein kinase
HER2: Human epidermal growth factor receptor-2
lv: Lentiviruses
MAPK: Mitogen-activated protein kinase
miR: miRNA, microRNA
mRNA: Messenger RNA
NC: Negative control
PR: Progesterone receptors
qRT-PCR: Quantitative real-time PCR
siRNA: Small interfering RNA
TNBC: Triple-negative breast cancer
UTR: Untranslated region
WT: Wild-type
