# Supplementary material for: MiR-25-3p promotes the proliferation of triple negative breast cancer by targeting BTG2
Source: Mol Cancer. 2018 Jan 8;17:4. doi: 10.1186/s12943-017-0754-0 (PMC5759260; doi:10.1186/s12943-017-0754-0)
Supplement: Additional file 1: Table S1. — Nine differentially expressed miRNAs from miRNA microarray assay in TNBC and adjacent normal tissues. (DOCX 126 kb) [file 12943_2017_754_MOESM1_ESM.docx]

**Additional file 1**

**Figure S1** miRNA profiling data obtained from fresh specimens of 5 paired triple-negative breast cancer specimens and adjacent normal tissues.

**
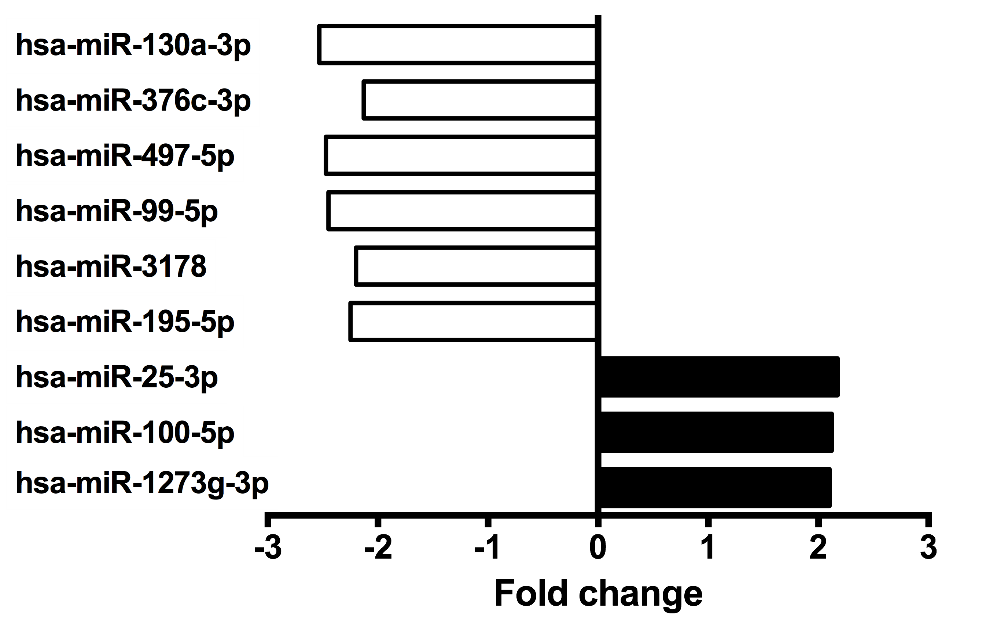
**

**Table S1** List of microarray prolifing data

|  |  | G1 |  | G2 |  |  |  |
| --- | --- | --- | --- | --- | --- | --- | --- |
| Reporter Name | p-value | Mean | StDev | Mean | StDev | Log2（G2/G1） | Fold change |
| hsa-miR-130a-3p | 3.60E-03 | 972 | 358 | 384 | 140 | -1.339850003 | 2.53125 |
| hsa-miR-376c-3p | 7.63E-03 | 700 | 232 | 329 | 101 | -1.089267338 | 2.127659574 |
| hsa-miR-497-5p | 8.48E-03 | 544 | 228 | 220 | 54 | -1.306103128 | 2.472727273 |
| hsa-miR-99-5p | 1.11E-02 | 4380 | 569 | 1790 | 729 | -1.290971282 | 2.446927374 |
| hsa-miR-3178 | 1.16E-02 | 3203 | 782 | 1457 | 668 | -1.136422921 | 2.19835278 |
| hsa-miR-195-5p | 1.68E-02 | 5361 | 1789 | 2384 | 967 | -1.169117899 | 2.248741611 |
| hsa-miR-25-3p | 1.77E-02 | 410 | 141 | 892 | 392 | 1.1214198 | 2.175609756 |
| hsa-miR-100-5p | 4.43E-02 | 336 | 114 | 713 | 382 | 1.085440844 | 2.12202381 |
| hsa-miR-1273g-3p | 4.76E-02 | 1045 | 380 | 2197 | 1142 | 1.072031927 | 2.102392344 |
