# Supplementary figures and images for: MiR-25-3p promotes the proliferation of triple negative breast cancer by targeting BTG2
Source: Mol Cancer. 2018 Jan 8;17:4. doi: 10.1186/s12943-017-0754-0 (PMC5759260; doi:10.1186/s12943-017-0754-0)

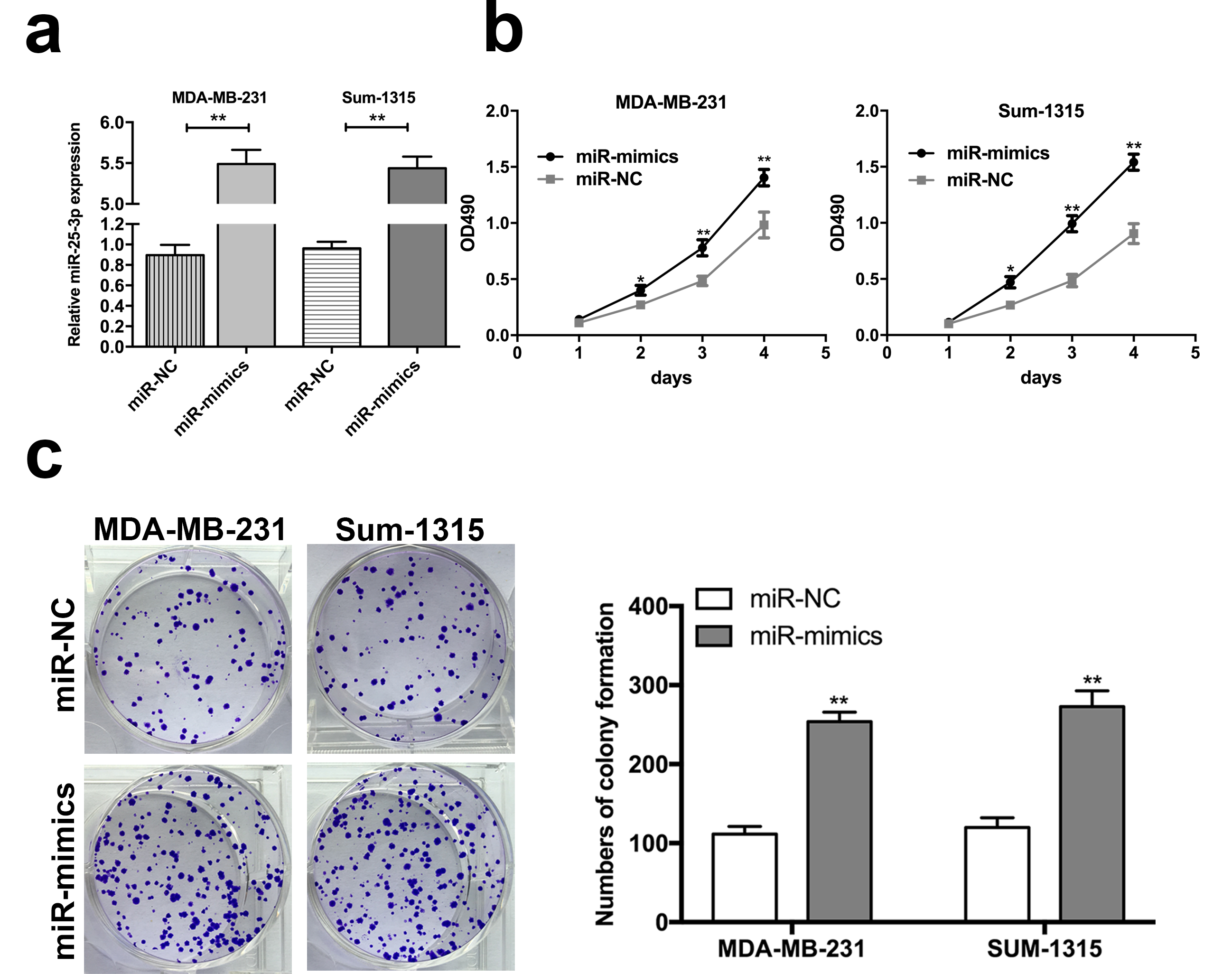

Supplement: Additional file 2: FigureS1. — a. qRT-PCR was used to verify the expression of miR-25-3p in MDA-MB-231 and Sum-1315 cells transfected with mimics b. Cell proliferation was determined by CCK-8 assays in MDA-MB-231, Sum-1315 cells transfected with miR-25-3p mimics. c. The colony formation results of cells transfected with mimics lentivirus. *p < 0.05, **p < 0.01. The data expressed as the mean ± SD. (TIFF 1901 kb) [file 12943_2017_754_MOESM2_ESM.tif]

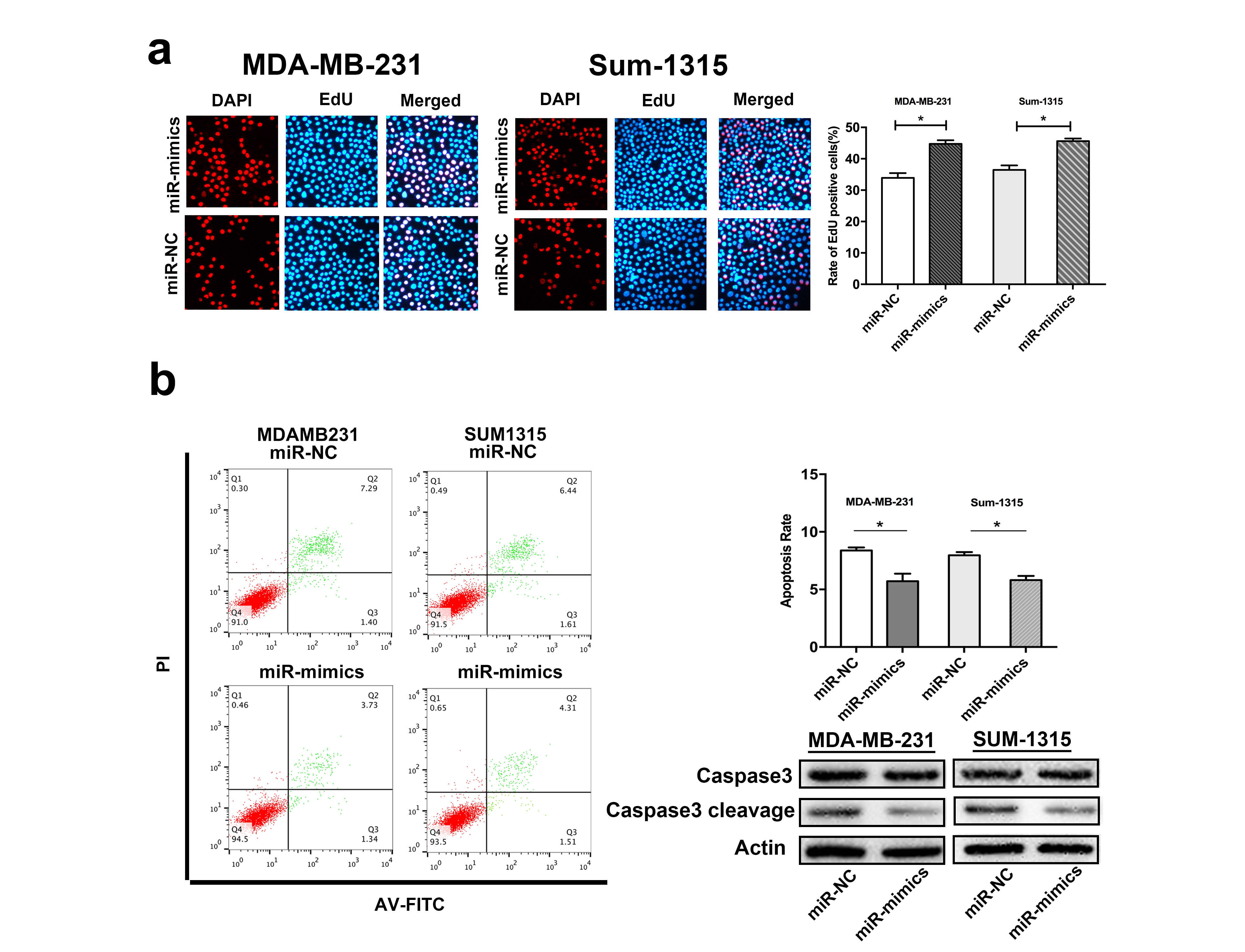

Supplement: Additional file 3: Figure S2. — a. Edu cell growth in MDA-MB-231, Sum-1315 after transfection with miR-25-3p-mimics compared with the control. b. Flow cytometry analysis of the effect of miR-25-3p expression alteration on cell apoptosis and apoptotic marker expression. *p < 0.05, **p < 0.01. The data expressed as the mean ± SD. (TIFF 3545 kb) [file 12943_2017_754_MOESM3_ESM.tif]
